# Letter to the Editor: Reaffirming Caution—The Unacceptable Vasoconstrictive Risk of CGRP‐Related Therapies in Moyamoya Angiopathy

**DOI:** 10.1111/ene.70481

**Published:** 2026-01-08

**Authors:** Charly Gaul, Steffen Naegel

**Affiliations:** ^1^ Headache Center Frankfurt Am Main Hesse Germany; ^2^ Medical Faculty University Hospital Essen Essen Germany; ^3^ Department of Neurology Alfried Krupp Krankenhaus Essen Ruettenscheid Essen Germany; ^4^ Department of Neurology University Hospital Halle Halle Germany

To the Editor,

With great interest we read the current consensus overview, “The Spectrum of Headaches in Moyamoya Angiopathy: From Mechanisms to Management Strategies ‐ A Consensus Review from the NEUROVASC Working Group” which provides a much‐needed framework for managing headaches in patients with Moyamoya angiopathy (MMA).

As neurologists specializing in headaches, we agree that both acute and prophylactic treatment of headaches in patients with MMA is very challenging. The stroke mechanisms in MMA are hemodynamic, embolic, and haemorrhagic, all of which carry different risks for treatment. High‐dose ASA, for example, may lead to an increased risk of bleeding in this patient group with a pre‐existing increased risk. NSAIDs are suboptimal due to their interaction with ASA used for ischemia prophylaxis; metamizole may lead to hypotension, and triptans, due to their vasoactive properties, are not only formally contraindicated in this context. Regarding prophylactic treatment, we find calcium channel blockers and beta blockers difficult due to hypotension and the resulting hemodynamic risks.

However, our most important addition to the Consensus Review is our stricter assessment of calcitonin gene‐related peptide (CGRP) antagonists. Although the authors adopted a cautious approach to the use of CGRP‐based therapies, we believe that the severity of the inherent vascular risk in MMA requires an even more definitive stance against these agents.

The available evidence, while limited, points to a potential for harm that far outweighs the therapeutic benefit in this high‐risk population.

## 1 Unacceptable Risk Profile of CGRP Blockade in MMA

The core pathophysiology of MMA involves progressive steno‐occlusive disease of the intracranial carotid arteries and the compensatory formation of fragile collateral (Moyamoya) vessels. In this delicate, flow‐compromised system, maintaining adequate cerebral blood flow (CBF) and oxygen delivery is paramount [1].

CGRP is one of the most potent endogenous vasodilators [2]. Its presence is particularly vital in the setting of ischemia, where it acts as a critical compensatory mechanism to maximize blood flow and perfusion pressure [3]. This appears to be of the utmost relevance when considering the pathophysiology of MMA. Blocking this compensatory mechanism carries an unacceptable theoretical risk of precipitating cerebral ischemic events. By dampening the body's intrinsic ability to induce vasodilation in areas of critical flow, CGRP‐related therapies could potentially shift the delicate balance, leading to hypoperfusion, transient ischemic attacks (TIAs), or stroke, particularly in patients with borderline flow dynamics.

The risk here is not just theoretical, as Mulder and colleagues were able to document very clearly and convincingly that CGRP‐blocking treatment leads to more severe strokes in animal models. Even transfer from bench to bedside is not straightforward. It appears reasonable that these data may also be applicable to humans [4].

The relevant RCTs with substances acting on the CGRP pathway excluded patients with any relevant vascular diseases. The consequence of this exclusion is evident in the product information. For example, the European product information (SmPC) for erenumab explicitly states that no safety data are available for patients with certain serious cardiovascular diseases [5].

Recent published data are not alarming; however, the results do not encourage us to neglect the topic [6, 7]. Data available from a population with increased vascular risk until now are based on single use of erenumab and do not deal with the courses of acute ischemic events [8]. Furthermore, even mechanisms of conventional myocardial infarction or ischaemic stroke are not an adequate model for the moyamoya disease. The mechanisms behind the moyamoya arteriopathy are not fully understood; however, these significantly differ from the pathologies in patients with arteriosclerosis.

The argument that these therapies should be considered because they are “highly effective” for migraine must be secondary to the principle of “First, do no harm.” There are no safety data and will likely never be due to ethical constraints to justify the use of agents with potent vasomotor effects in a primary cerebral vasculopathy. Relying on safety data from the general migraine population, who do not possess the unique compensatory flow challenges of MMA, is inappropriate and misleading.

## 2 Call for Contraindication

As they impair the urgently needed compensatory vasodilation, CGRP‐related therapies (monoclonal antibodies and gepants) should also be classified as contraindicated for MMA patients, similar to triptans in acute treatment.

Headache management in MMA must prioritize treatments that are neutral with respect to vascular tone. While the management of refractory migraine remains a challenge, the potential for drug‐induced ischemia in MMA constitutes a severe, life‐threatening complication that outweighs the goal of headache relief.

We urge the adoption of clear guidelines that caution against the use of CGRP‐related therapies in MMA, reinforcing that the risk is, in this specific clinical context, a highly probable and potentially catastrophic hazard.

## Author Contributions

**Steffen Naegel:** conceptualization, writing – original draft, writing – review and editing. **Charly Gaul:** conceptualization, writing – review and editing.

## Disclosure

Declaration of AI Technologies: During the preparation of this work, the authors used DEEPL to improve language and readability of parts of the text. After using this tool, the authors reviewed and edited the content as needed and took full responsibility for the content of the publication.

## Ethics Statement

The authors have nothing to report.

## Consent

The authors have nothing to report.

## Conflicts of Interest

Charly Gaul has received honoraria for consulting and lectures within the past 3 years from Abbvie, Lilly, Novartis Pharma, Hormosan Pharma, Sanofi‐Aventis, Lundbeck, Perfood, Vectura Fertin Pharma, Chordate, Pfizer, Dr. Reddys, Merz, Reckitt‐Benckiser, Organon, Orion, and TEVA. His research is supported by a grant from the German Research Foundation (DFG). He does not hold any stocks in pharmaceutical companies. Steffen Naegel has received honoraria for consulting and lectures within the past 3 years from Lilly, Novartis Pharma, Hormosan Pharma, Lundbeck, and TEVA.

## Data Availability

Data sharing not applicable to this article as no datasets were generated or analysed during the current study.
